# Fluorescence Signal-Readout of CRISPR/Cas Biosensors for Nucleic Acid Detection

**DOI:** 10.3390/bios12100779

**Published:** 2022-09-20

**Authors:** Zhaohe Huang, Sitong Liu, Xiaojing Pei, Shujing Li, Yifan He, Yigang Tong, Guoqi Liu

**Affiliations:** 1 Institute of Cosmetic Regulatory Science and College of Chemistry and Materials Engineering, Beijing Technology and Business University, Beijing 100048, China; 2 College of Chemistry and Materials Engineering, Beijing Technology and Business University, Beijing 100029, China; 3 Biotecnovo (Beijing) Co., Ltd., Beijing Economic and Technological Development Zone, Beijing 100176, China

**Keywords:** CRISPR, fluorescence, nucleic acids detection

## Abstract

The CRISPR/Cas system is now being used extensively in nucleic acid detection applications, particularly after the trans-cleavage activity of several Cas effectors was found. A CRISPR/Cas system combined with multiple signal-readout techniques has been developed for various molecular diagnostics applications. Fluorescence is now a widely utilized dominant read-out technique in CRISPR biosensors. An in-depth understanding of various fluorescence readout types and variables affecting the fluorescence signals can facilitate better experimental designs to effectively improve the analytical performance. There are the following two commonly used types of CRISPR/Cas detection modes: the first is based on binding activity, such as Cas9 and dCas9; the second is based on cleavage activity, such as Cas12a, Cas12b, Cas13, and Cas14. In this review, fluorescence signal-readout strategies from the last 5 years based on the binding activity and cleavage activity of the CRISPR/Cas system with fundamentals and examples are fully discussed. A detailed comparison of the available fluorescent reporter sequences and design principles is summarized. Current challenges and further applications of CRISPR-based detection methods will be discussed according to the most recent developments.

## 1. Introduction

Nucleic acid is the carrier of genetic information and can be used as a biomarker for many disease diagnoses. Most existing nucleic acid assays rely on amplification of nucleic acid, with high cost, low throughput, and complex operations. Clustered Regularly Interspaced Short Palindromic Repeats (CRISPR) and CRISPR-associated (Cas) system is an acquired immune system found in most bacteria and all archaea [1,2]. Since its initial discovery, the number of different CRISPR–Cas systems has developed speedily. The CRISPR/Cas system contains two classes (class 1 and class 2) and six subtypes [3]. Cas effectors from class 2, such as type II (Cas9), type V (Cas12), and type VI (Cas13), are commonly used in genome editing because they can efficiently conduct both target identification and cleavage activities with a single protein [4,5,6,7]. It shows great potential in nucleic acid detection, which relies on CRISPR RNA (crRNA) or a single-guide RNA (sgRNA) to direct Cas effector proteins to bind to specific nucleic acid sequences and process them or other nucleic acid sequences, such as binding or cleavage [1,2,8,9]. Cas9, dCas9, Cas12a, Cas13a, and Cas14 are the most often employed Cas effectors and play a vital role in molecular diagnostics applications. By modifying the sequence of crRNA, any target nucleic acid can be easily detected. The CRISPR/Cas system has been developed in collaboration with a range of signal readout methods for diverse target assays due to the precise binding activity and powerful cleavage activity of the CRISPR/Cas system [10,11,12,13]. The Cas9 includes HNH and RuvC-like nuclease domains, which each cleave one target double-strand DNA (dsDNA) via the following two RNA components: mature crRNA and trans-activating crRNA (tracrRNA) [14,15,16]. Furthermore, sgRNA was found to direct Cas9 binding and cleavage by combining the sequences of crRNA and tracrRNA [17,18]. The protospacer adjacent motif (PAM) sequence, which is present in both complementary and non-complementary DNA strands, is the location of the site-specific cleavage, which occurs in three base pairs upstream [5]. dCas9, the catalytically inactive Cas9, lacks cleavage activity compared to Cas9 by suppressing two mutations in the structural domains of RuvC1 and HNH nucleases [15]. The dCas9/sgRNA complex, on the other hand, preserves great specificity for target DNA and has been employed as a nickase in biosensors [19]. Unlike Cas9, Cas12a recognizes target dsDNA using a short T nucleotide-rich PAM sequence [12]. A single RuvC domain is enough to cleave both strands of target DNA using a single crRNA as a guide, producing a PAM-distal dsDNA break with staggered 5’ and 3’ ends [20]. Additionally, Cas12a may also completely disintegrate non-specific single-strand DNA (ssDNA) after binding to a target, a process known as “trans cleavage”. Cas13a, such as Cas12a, possesses trans cleavage ability for non-specific nucleic acids after attaching to target RNA [21,22]. Yet, Cas13a completely cuts non-target RNA after forming the guide-target RNA duplex and employs two conserved HEPN domains to cut target RNA at uracil sites [11]. Furthermore, rather than a PAM sequence, Cas13a detects the target RNA through a 3’ protospacer flanking site (PFS) [23]. Unlike the PAM-proximal seed area required by dsDNA-targeting and the PAM-distal sequence recognized by Cas12a, Cas14’s identification is mediated by interactions around the center of the ssDNA target. Similar to Cas12a, Cas14 detects a target and initiates non-specific ssDNA trans-cleavage activity [10]. The Cas effectors listed above may bind the target nucleic acids with a 20–30 bp long guide RNA (sgRNA or crRNA). Additionally, PAM sequence recognition is essential for Cas effectors to recognize target dsDNA, causing the target dsDNA helix to unwind and enabling the guide RNA to hybridize it [24].

**Figure 1:**
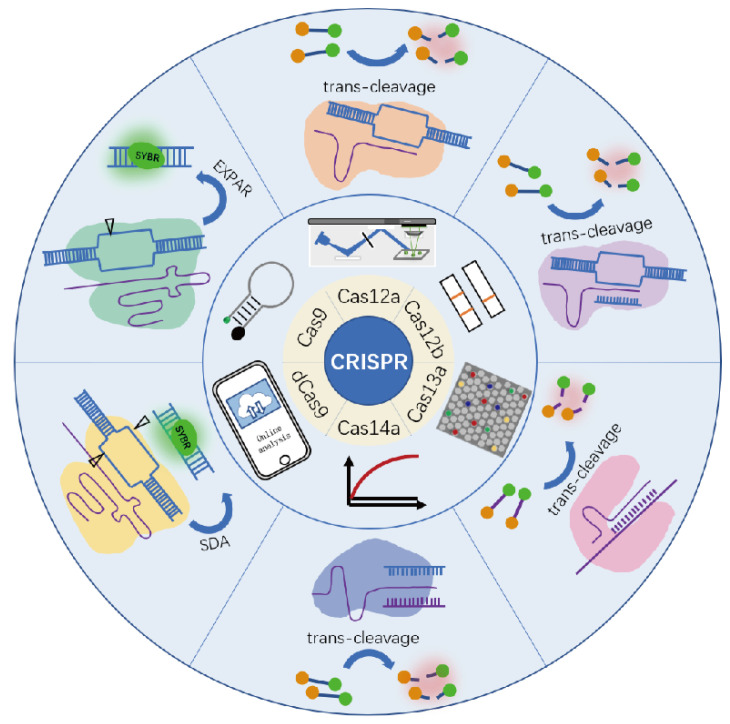
Various commonly used CRISPR system components, signal read-out mode, and detection method.

A reliable integrated biosensor should be capable of identifying a target of interest as well as signal transduction for readout. Although CRISPR/Cas-based biosensors have been summarized in some recent reviews. For example, Kaminski et al. summarized various Cas enzymes in clinical diagnostic tests, and Wang et al. reviewed the signal amplification and output of CRISPR/Cas-based biosensing systems [25,26]. The signal readout of CRISPR/Cas has never been classified systematically and discussed comprehensively. In general, CRISPR/Cas biosensors can transduce signals in a variety of ways, such as various optical and electrochemical signals [19,27,28,29,30,31,32,33]. Fluorescence is widely used because of the outstanding advantages in qualitative and quantitative when compared to other methods. In this mini-review, we emphasize the fluorescence signal-readout strategies based on the binding activity and cleavage activity of commonly used CRISPR/Cas systems from fluorescent intensity signals, fluorescent digital signals, fluorescent nanomaterials assisted signals, fluorescent naked eye signals, and others. Figure 1 shows the components of these CRISPR/Cas systems and the identification and cleavage methods, as well as the signal read-out techniques. For each Cas effector, we listed some typical signal transduction categories and main features with representative work (Table 1). The difficulties and challenges currently encountered in applying CRISPR/Cas systems to the assay process will also be discussed.

## 2. Fluorescence Intensity Signals

The fluorescence intensity-based signal readout is easy to encode and provides a simple and effective way for CRISPR biosensors. Most of its analytical signals are obtained based on real-time fluorescence intensity measurements, which describe the average behavior of a large number of molecules over a period of time. Fluorescent probes, such as fluorophores, quantum dots (QDs), nanoclusters, carbon dots, and fluorescent nanomaterials, which display either increased or quenched fluorescence intensity in response to the target objects.

The detection methods used by different Cas proteases based on binding and cleavage activity employing fluorescent molecules as signal labels by measurement of fluorescence intensity will be the main topic of this section.

### 2.1. Binding Activity—Cas9, dCas9

Cas9 and dCas9/sgRNA complexes maintain high specificity for target DNA and have been employed in biosensors and molecular diagnostics. Cas9 is able to recognize and cut the dsDNA. The sgRNA first finds a specific PAM sequence (5′–NGG–3′) in a non-target DNA strand, distant 10–12 nucleotides of the PAM. The target and non-target DNA strands are bound by HNH and RuVC responsible for the cleavage activity, respectively. The deactivated enzyme dCas9 works as a recognition element, specifically binding to the target DNA sequence without cutting it [1,56,57,58]. Metal-organic framework (MOF), which are nonporous materials having structures based on classical coordination bonds between metal cations and electron donors, which has been used for fluorescence quenching/recovery by adsorption/desorption of fluorophore-labeled ssDNA. Sun et al., used the MOF structure (UiO66) to develop an SDA-RCA amplification method with CRISPR/Cas9 for fluorescence detection of *E. coli* [35]. Following the recognition and cleavage of a one-strand nick of target DNA by two Cas9 sgRNA complexes, strand displacement synthesis expanded at the nick and displaced the original DNA strand. Pre-incorporation of a fluorescent probe bound to UiO66 into the system. When the long ssDNA binds to the probe, it separates them from UiO66, and the quenched fluorescence is recovered. As a result, the recovery fluorescence intensity can be used to quantify the target DNA.

**Figure 2:**
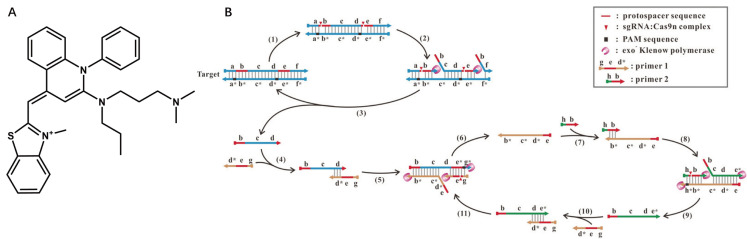
(**A**) Structure of SYBR Green I; (**B**) the scheme of the proposed Cas9nAR for amplification of a DNA fragment of from genomic DNA. Reprinted with permission from ref. [19], copyright (2019) John Wiley and Sons.

There are fluorescent dyes that bind specifically to dsDNA, such as SYBR Green I and ethidium bromide. A common feature of these dyes is that they are not fluorescent unless incorporated into the backbone of dsDNA. As the amount of dsDNA increases during the reaction, the fluorescent signals increase correspondingly. With the SYBR Green I for noncovalent DNA staining, Huang et al. created a CRISPR/Cas9 induced exponential amplification approach (CAS-EXPAR) that combines the benefits of Cas9/sgRNA site-specific cleavage and the rapid amplification kinetics of EXPAR (Figure 2) [34]. Sensitive DNA detection with CAS-EXPAR could be achieved in 1 h by combining a real-time fluorescence intensity analysis approach. A dCas9-based amplification process, developed by Wang et al., is a simple but innovative nucleic acid amplification approach (Cas9nAR) [19]. The intercalation dye SYBR Green I was used to track Cas9nAR progression in real-time, and the fluorescence intensity is proportional to the concentration of dsDNA products.

### 2.2. Cleavage Activity—Cas12, Cas 13, Cas14

It is found that RNA-guided DNA binding triggers Cas12a’s indiscriminate trans-cleavage activity [12]. Cas13a, such as Cas12a, possesses the trans-cleavage ability for non-specific nucleic acids after binding to target RNA [11]. Yet, Cas13a completely cuts non-target RNA following the creation of the guide-target RNA duplex. Similar to Cas12a, Cas14 detects a target and initiates non-specific ssDNA trans-cleavage activity [10]. Based on this principle, fluorescence resonance energy transfer (FRET) reporters with one fluorophore acting as an energy source or fluorophore and the other as a receptor at both ends of ssDNA or RNA are designed in CRISPR biosensors [59,60,61]. It begins to cleave these FRET reporters non-specifically when Cas enzymes start trans-cleavage activity due to the recognition of targets. As we all know, with one fluorophore acting as an energy source or fluorophore and the other as a receptor, FRET is a distance-dependent mechanism of energy transfer between fluorophores and quenchers [62]. The length of the fluorophore/quencher labeled ssDNA reporter is an important factor affecting analytical performance [63,64]. When ssDNA reporters are cleaved by Cas enzymes, the reporter dye is separated from the quencher dye. The quenching effect is gone, and thus reporter dye fluorescence will be detected by the instrument. The released reporter dye signal is proportional to the concentration of targets. The DNA endonuclease-targeted CRISPR trans-reporter (DETECTR) approach provides attomolar sensitivity for DNA detection by combining Cas12a ssDNase activation with isothermal amplification. Li et al. employed PCR or other isothermal amplification techniques to specifically amplify the target DNA [31]. The amplicon was then combined with the Cas12a/crRNA complex, and a ternary complex was formed when the target DNA was present. The quenched fluorescent ssDNA reporter was trans-cleaved upon the formation of the ternary complex, releasing the fluorescence.

In contrast to Cas12a, which had a greater nonspecific ssDNA trans-cleavage rate with target dsDNA than with target ssDNA, Cas12b demonstrated a distinct target preference in trans-cleavage. By using the Cas12b, a thermophilic RNA-guided endonuclease from the type V-B CRISPR/Cas system, Li et al., created HOLMESv2 for discriminating single nucleotide polymorphism (SNP) [48]. It was also combined the nucleic acid amplification and target identification phases into a single system to streamline operations and prevent cross-contamination. The needed target concentration was reduced to 10^−^^8^ nM when paired with loop-mediated isothermal amplification (LAMP), which was equivalent to Cas12a.

**Figure 3:**
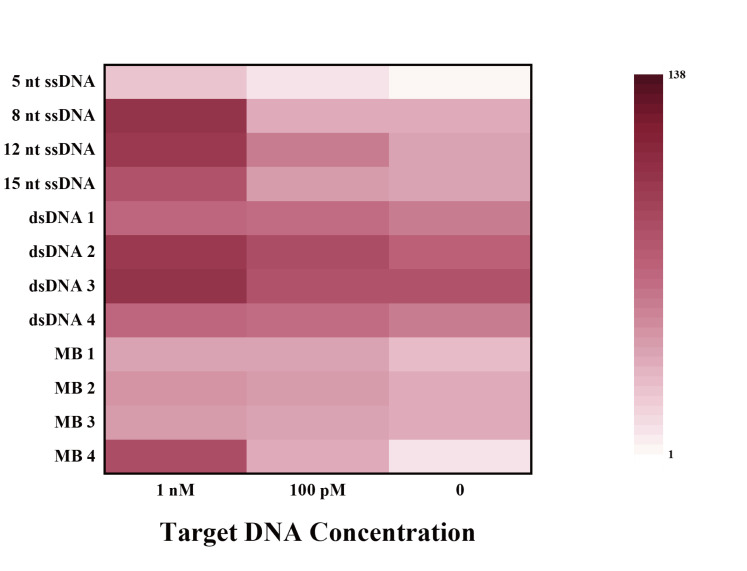
Fluorescence ratios of different types of FRET reporters at different target concentrations. Reprinted with permission from ref. [65], Copyright (2022) Elsevier B.V.

The FRET reporters used in Cas12a studies include 5(6)-carboxyfluorescein (FAM) as the donor and BHQ1 as the quencher. Although the ssDNA reporters are commonly used for research and applications (Table 2), limiting selections of ssDNA reporters from an application and probe diversification standpoint. We recently investigated the properties of various types of fluorescent probes for CRISPR in detail (Figure 3) [65]. We demonstrated that the trans-cleavage of Cas12a is not limited to ssDNA or dsDNA reporters but can be extended to molecular beacons (MB). MBs are a class of small, single-stranded oligonucleotides with a fluorophore and a quencher at the two termini, which have been widely used as hybridization-activated FRET probes but rarely used in CRISPR-based fluorescent assays. On the 5′ and 3′ termini of 37-nt ssDNA, we labeled the MB reporter with Taxes Red and BHQ2. A secondary structure such as the hairpin shape (15-nt in the loop structure and 12-bp in the stem structure) is formed after annealing. Results demonstrated that MB probes can achieve better analytical performance than ssDNA and dsDNA probes and that FRET probes modified with a fluorophore (Texas Red) are more sensitive than modified FAM. Accordingly, we developed a highly sensitive SARS-CoV-2 detection with a sensitivity as low as 100 fM, which was successfully achieved without an amplification strategy. A real coronavirus, GX/P2V instead of SARS-CoV-2, was chosen for practical application validation. After magnetic bead-based rapid RNA extraction and RT-PCR amplification, a minimum of 2.7 × 10^1^ copies/mL can still be obtained. The inspiration can also apply to other Cas effectors with trans-cleavage activity, which provides perspectives for simple, highly sensitive, and universal molecular diagnosis in various applications.

**Figure 4:**
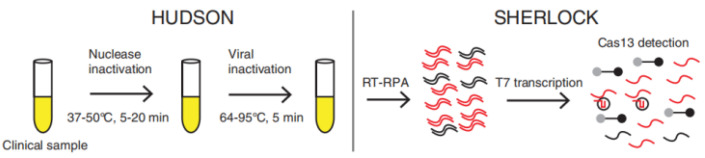
Schematic of direct viral detection by HUDSON and SHERLOCK. Reprinted with permission from ref. [22], Copyright (2018) The American Association for the Advancement of Science.

Numerous efforts have been devoted to RNA virus detection based on the trans-cleavage activity of RNA reporters of Cas13a after target RNA recognition (Table 3). For example, Gootenberg et al. designed a Cas13a-mediated in vitro nucleic acid detection platform named Specific High-Sensitivity Enzymatic Reporter UnLOCKing (SHERLOCK), which uses commercially available RNA fluorescent probes as fluorescent signals [21]. The dsDNA samples or RNA samples were amplified by recombinase polymerase amplification (RPA) or RT-RPA, and then T7 RNA polymerase transcribes the amplified DNA to RNA. The target RNA products trigger the trans-cleavage activity of Cas13 and cause the fluorescent reporters to be cleaved to release fluorescent signals. Sherlock Biosciences, Inc. (Watertown, MA, USA) has received EUA (Emergency Use Authorization) approval in the United States for a fluorescent CRISPR kit. RNA is extracted from clinical samples using the PureLink™ Viral RNA/DNA Mini Kit. The viral RNA is then reverse transcribed and amplified by RT-LAMP, which activates the cleavage activity of the CRISPR complex to cut reporters, thereby releasing a fluorescent light signal that can be observed by the plate reader. To make the actual operation easier, Myhrvold et al., developed HUDSON, a method to lyse viral particles and deactivate the large amounts of ribonucleases found in body fluids using heat and chemical reduction, to identify viral nucleic acid directly from bodily fluids via SHERLOCK (Figure 4) [21,22]. Without dilution or purification, HUDSON-treated urine or saliva can be introduced directly into the RPA reaction mixture. Despite lower viral titers than those in serum, HUDSON and SHERLOCK enabled sensitive and specific ZIKV nucleic acid detection in 1 h.

The Cas14 system is the smallest functional CRISPR system discovered to date, about one-third the size of Cas9, and features PAM sequence independence and high specificity and fidelity during shearing. Harrington et al. used Cas14 to design the DETECTR platform by combining the isothermal amplification method of RPA with the high-fidelity detection of DNA by Cas14 [10]. The amplified target DNA induced Cas14 to cleave the fluorescent reporter ssDNA, resulting in fluorescent signal recovery. When Cas14a was incubated with various sgRNA or target ssDNA, it showed a strong preference for longer ssDNA fluorescent substrates. The 12 T-base ssDNA reporter was the first to show the strongest fluorescent signal [70]. Wei et al. were the first to discover that Cas14a has trans-cleavage of ssDNA, specifically activated by a target RNA, without self-destruction [28]. Accordingly, they created a series of target RNA and ssDNA with varying lengths and mutation sites to investigate the effect of the active factor (target RNA or ssDNA) on Cas14a’s trans-cleavage activities. It was discovered that the Cas14a/sgRNA-target RNA self-assembled complex can only trans-cleave the ssDNA reporter (Table 4). Furthermore, when triggered by target RNA, the substrate selectivity of this complex can be higher than when activated by target ssDNA. Based on this finding, a new ATCas-RNA platform was created that can detect pathogens using a FRET reporter with excellent selectivity and sensitivity, similar to the Cas13-based RNA diagnostic system.

## 3. Fluorescent Digital Signals

It is a continuous challenge to improve the detection limit and sensitivity of assays for capturing and properly estimating the concentration of a certain target. Traditional fluorescent methods for concentration measurement are based on a bulk solution response. The digital biosensor is a game-changing technique for both analytical chemistry and single-molecule studies. The most notable distinction between digital bioassays and other analytical methods is that the former counts just the number of a binary (positive or negative) signal from a vast population of individual reactors for concentration determination, whereas the latter records the absolute signal strength from a single reactor. Digital bioassays work by separating reaction solutions into micrometer-sized compartments [71]. In digital bioassays, rather than quantifying the absolute intensity of ensemble signals from tubes or microtiter plates, just the fraction of microcompartments displaying positive signals is tallied. The bulk solution enzyme concentration is calculated by dividing the enzyme-containing solution and a suitable substrate into a large number of femtoliter-sized reactor containers. The reactor volumes are small enough that they can only hold zero or one enzyme molecule. A binary readout approach can be used to count enzyme molecules by observing the presence or absence of a fluorescent product arising from single enzyme molecule catalysis in each reaction vessel.

**Figure 5:**
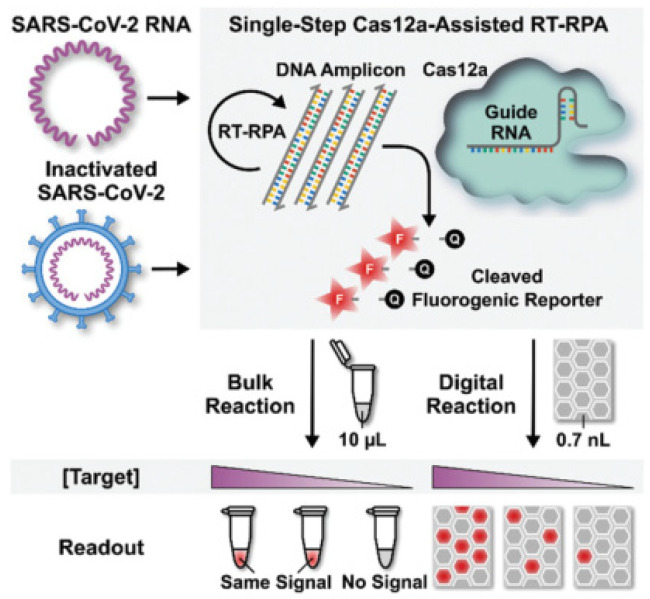
Schematic of digital decovid assay. Reprinted with permission from ref. [44], Copyright (2020) John Wiley and Sons.

Digital biosensors combined with excellent CRISPR enzyme properties would create new sparks. Park et al. designed and applied the first digital CRISPR/Cas-assisted assay—digitization-enhanced CRISPR/Cas-assisted one-pot virus detection (deCOViD)—to SARS-CoV-2 detection (Figure 5) [44]. The DeCOViD was achieved by adjusting and discretizing a one-step CRISPR/Cas12a-assisted RT-RPA into 0.7 nL digital reaction wells within commercially available microfluidic digital chips. With a high signal-to-background ratio, broad dynamic range, and high sensitivity, deCOViD can achieve qualitative detection in 15 min and quantitative detection in 30 min using evenly elevated digital concentrations. SARS-CoV-2 RNA and inactivated SARS-CoV-2 can be fluorescently identified in deCOViD using a single-step technique that combines RPA and CRSIPR/Cas12a-based detection. RNA targets are reverse transcribed and amplified into DNA amplicons via RT-RPA, which activates Cas12a guide RNA complexes, which cleave ssDNA fluorogenic reporters and produce fluorescence. This single-step assay is then fine-tuned to guarantee that it can be loaded quickly onto commercially available microfluidic digital chips and discretized reliably within digital reaction wells. During assay digitization, each copy of the target is separated at a locally elevated concentration inside digital reaction wells, allowing for rapid amplification regardless of sample concentration. This beneficial method improved the signal-to-background ratio, dynamic range, and sensitivity.

**Figure 6:**
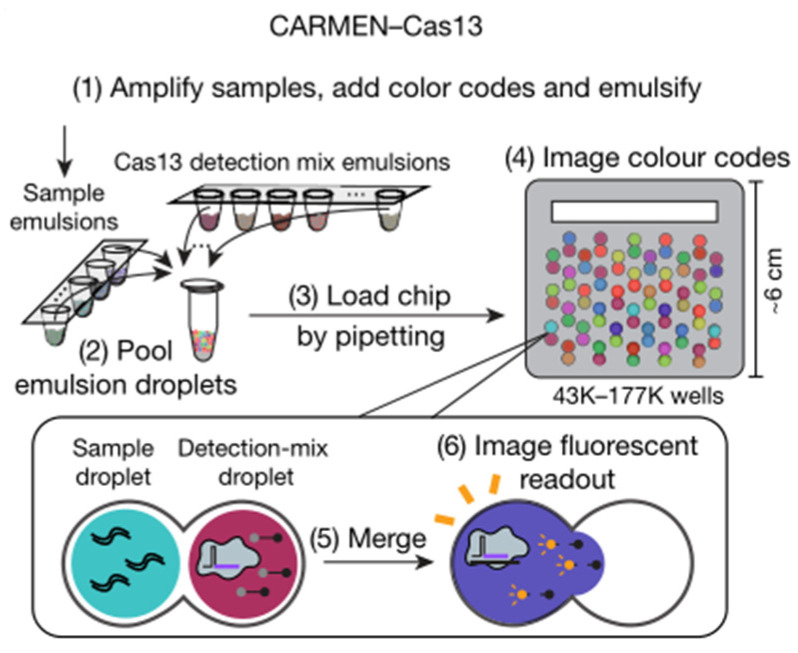
Schematic of CARMEN–Cas13 workflow. Reprinted with permission from ref. [53], Copyright (2020) Springer Nature.

One-pot digital warm-start CRISPR (WS-CRISPR) reaction was partitioned into sub-nanoliter aliquots using QuantStudio 3D digital chips to create the digital WS-CRISPR assay for detecting SARS-CoV-2 in clinical COVID-19 samples with high sensitivity by Ding and coworkers [47]. The WS-CRISPR reaction combined low-temperature reverse transcription dual-priming isothermal amplification (RT-DAMP) and CRISPR/Cas12a-based fluorescence detection in a one-pot format and is efficiently initiated at temperatures above 50 °C, preventing premature target amplifications at room temperature and allowing accurate nucleic acid digital quantification. In one tube, a one-pot WS-CRISPR reaction mixture was first prepared. Over ten thousand sub-nanoliter (0.7 nL) microreactions are isolated in microwells after being dispersed into the QuantStudio 3D digital chip. Each microreaction with SARS-CoV-2 RNA target undergoes WS-CRISPR reaction and generates intense green fluorescence (positive spots) when incubated at 52 °C, whereas those without target do not (negative spots). The digital WS-CRISPR assay was designed to quantitatively identify 32 clinical swab samples and three clinical saliva samples by targeting the SARS-CoV-2 nucleoprotein (N) gene. Furthermore, the digital WS-CRISPR test can detect SARS-CoV-2 in heat-treated saliva samples without the need for RNA extraction. Digital bioassays are compatible with existing classical techniques widely used in normal biological, chemical, or clinical laboratories. Ning et al. described a COVID-19 CRISPR-FDS assay that does not require any specific equipment beyond what is commonly available in research and clinical settings [45]. RNA extraction, target amplification, and fluorescence signal detection are the three processes of CRISPR-FDS. On a 96-well half-area plate, the gRNA/Cas12a complex, which is regulated by a target-specific synthetic gRNA, recognized the target amplicon, and caused it to non-specifically cleave a reporter oligo modified with fluorescein and a quencher molecule at each terminal, resulting in the release of fluorescence. Combined CRISPR/Cas13-based RNA recognition with microchamber-array technologies, Shinoda et al., developed a CRISPR-based amplification-free digital RNA detection named SATORI [51]. Amplification-free RNA molecular detection at the single-molecule level was achieved by using a device containing more than 1,000,000 through-holes in a femtoliter microchamber. The microchamber device is a glass block with an intake port mounted to a glass substrate with 1,000,000 microchambers, with a U-shaped spacer seal in between. The pre-assembled Cas13-crRNA complexes were mixed with FRET reporters, target RNA, and loaded into the microchamber device that were used in SHERLOCK. After the sealing of the device, a fluorescent signal released from the cleaved FRET reporters can be observed by fluorescence microscopy. Furthermore, Ackerman et al. developed a highly multiplexed nucleic acid detection platform called Carmen (Figure 6) [53]. Each nucleic acid sample is amplified by RPA or PCR and combined with a unique, solution-based fluorescent color code that acts as an optical identifier. In the fluorous oil, each color-coded solution was emulsified to produce 1 nL droplets. The Cas13-detection mix, which contained commercially available quenched fluorescent RNA reporter, a sequence-specific crRNA, and Cas13, was operated in the same way as the nucleic acid sample. The microwell-array chip is made of polydimethylsiloxane (PDMS), which has a large number of microwells that each of them can hold only two droplets. A variety of different droplets can be mixed, and after two droplets are randomly filled in the microwells of the chip, the contents of the two droplets added to the microwells can be determined by observing and identifying the color code of the droplets through a fluorescence microscope. If the nucleic acid sample in the droplet triggers the trans cleavage activity of Cas13, it will cause the RNA reporter to be cut and release fluorescence, and the corresponding detection result can be derived from the color of the mixed-droplet.

## 4. Fluorescent Nanomaterials Assisted Signals

Nanomaterials have distinct physical and chemical characteristics, making them ideal candidates for the development of innovative fluorescent biosensors. The enormous potential of such fluorescent nanomaterials has opened the way for the development of novel biomolecule assays with high analytical capabilities, such as sensitivity, cost-effectiveness, and simplicity of use. Various fluorescent nanomaterials with excellent fluorescence characteristics, such as QDs, photonic crystals (PHC), and metal nanoparticles, have been widely employed for CRISPR biosensors. In this section, we will focus on the detection methods of different Cas enzymes using fluorescent nanomaterials as signal labels through CRISPR fluorescent measurement.

### 4.1. Quantum Dot

QDs, as fluorescent semiconductor nanocrystals, have excellent photoluminescent quantum yields and great photochemical stability. They feature broad absorption and a narrow and symmetric photoluminescence spectrum (from UV to near-infrared) that may be modified by altering the size and chemical composition of the nanocrystals. The excellent optical properties make QDs the ideal candidates for CRISPR biosensors. In this context, Zhou et al., developed a CRA-LFB assay using a new CRISPR/Cas12a-based fluorescence enhanced lateral flow biosensor (LFB) in combination with functionalized QDs and recombinase-assisted amplification (RAA) [72]. The Cas12a-mediated trans-cleavage activation generated by the target DNA to digest biotin-DNA probes was seen in the CRA-LFB assay, which had no complementarity with the capture probe mounted on the test (T) line and resulting in an undetected T line fluorescence signal on LFB. The fluorescence intensity of the T and control lines could be measured using the naked eye or a fluorescence strip reader. This assay can detect *S. aureus* in both spiked and natural meat and vegetable samples.

### 4.2. Photonic Crystal Barcodes

Photonic crystal barcodes (PhC) are artificial periodic dielectric structures with photonic bandgap properties and unique optical phenomena such as suppressed spontaneous emission, highly reflective omnidirectional mirrors, and low-loss waveguides. Due to its exceptional encoding stability and resistance to interference from fluorescent background or photo-bleaching, PhC barcodes, which may be created via the self-assembly of monodisperse colloidal nanoparticles, have caught the attention of scientists. These properties make PhC barcodes a promising platform for CRISPR biosensors without disrupting the fluorescent signal. For example, Zhang et al. developed a CRISPR/Cas9 technology for multiplex and sensitive nucleic acid detection based on bioinspired PhC barcodes [41]. Because of the polydopamine (PDA) coating, the bioinspired PhC barcodes feature not only discrete structural color as encoding components but also rich functional surface groups for probe immobilization. The CRISPR/Cas9 system detected and cleaved target DNA, generating ssDNA with the assistance of the Klenow fragment, which was subsequently captured by PhC barcodes, and the detecting signal was amplified by a hybridization chain reaction (HCR). Different colors of colloidal crystal barcodes were employed for multiplexed detection, with the fluorescence signal only appearing on the colloidal crystal particles capturing the matching color. It was able to detect Human Papilloma Virus (HPV) nucleic acids with a sensitivity limit of 0.025 pM and be multiplexed assayed with high accuracy and specificity, providing a fresh insight for multiplexed biomarker quantification, and showing great potential in clinical disease diagnostics.

### 4.3. Metal Nanoparticles

Nanometallic materials are metals and alloys that form nanocrystalline grains. It has the characteristics of a grain boundary ratio, specific surface energy, and a large ratio of surface atoms. Noble metal nanomaterials (NMN) benefit from localized surface plasmon resonance (LSPR), which can be used as a highly sensitive signal conversion unit, thus enabling highly sensitive assay. When the distance between the fluorophore and the NMNs is less than about 2 nm, NMNs can function as fluorescence quenchers. This property can be used to improve the primary signal intensity when detecting biomaterials with NMNs such as Au and Ag nanomaterials.

**Figure 7:**
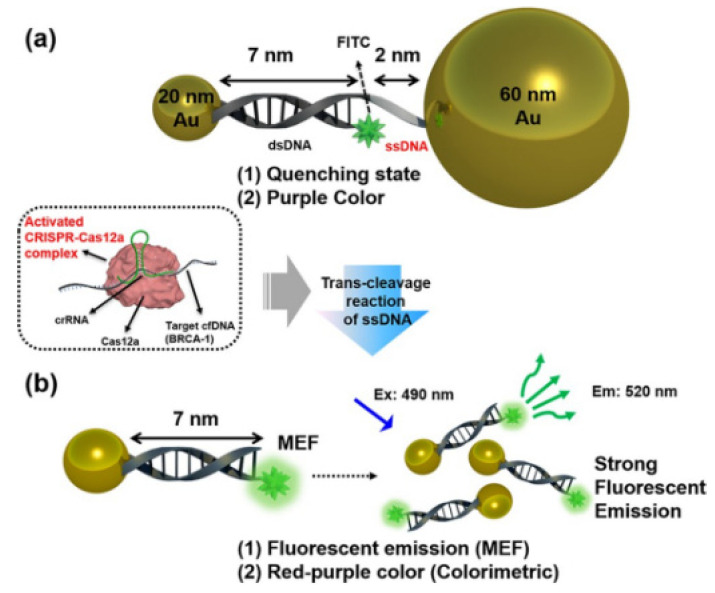
Schematic diagram illustrating our proposed cell-free DNA (cfDNA) detection method using DNA-functionalized Au nano-particles via colorimetric- and metal-enhanced fluorescence (MEF). Reprinted with permission from ref. [30], Copyright (2020) American Chemical Society.

Utilizing DNA-functionalized Au nanoparticles (AuNPs) and metal-enhanced fluorescence (MEF) effects, Choi et al. developed a cfDNA detection biosensor based on CRISPR/Cas12a nucleic acid amplification (Figure 7) [30]. MEF happened when the target cfDNA activated the Cas12a complex, followed by ssDNA degradation between AuNP and fluorophore, resulting in color shifts from purple to red-purple. Breast cancer gene-1 was detected with great sensitivity in 30 min with this technique. This rapid and highly selective sensor could be utilized in POCT scenarios to assess nucleic acid indicators such as viral DNA.

## 5. Portable Fluorescent Signal Readout

Since fluorescence needs an excitation light source, portable signal reading is not easy to achieve in general. Recently, many researchers have developed small fluorescence spectrometers for CRISPR biosensors by using a laser or LED as a light source and the naked eye or smartphone as a detector, which is convenient for point-of-care (POC) tests [73,74,75]. Naked-eye detection can get rid of the use of large instruments and the involvement of professionals and does not even require specialized skills training. The test can be performed under very simple conditions and the results can be known by the change of color. Fluorescence-based CRISPR/Cas systems still require instruments to read out the signal and do not allow true naked-eye testing. However, by exploiting the special optical properties of gold nanoparticles and endowing them with certain functions, naked-eye detection of CRISPR/Cas systems can be achieved [30]. In addition, smartphones, which are almost a must-have, are also a convenient option for signal readout. The combination of smartphones and cleverly designed microdevices with CRISPR/Cas technology greatly enhances the convenience and safety of detection, reducing the difficulty of the operation and the risk of centralized cross-infection [76]. In the statistics, summary, query, and other aspects of data also have a huge potential for development and broad application prospects. Using a fluorescence-based POC system to combine potent CRISPR/Cas assay with a fluorescence-based POC system for speedy and accurate virus detection, He et al., reported a method for African swine fever virus (ASFV) target DNA detection [77]. The POC system has a fluorescence sensing device with a 488 nm laser as an excitation source by self-designed compact detection equipment. The small fluorescence-sensing unit is aligned with an 80× disposable cartridge, thus matching the high-throughput of commercialized PCR systems. The Cas12a/crRNA/ASFV DNA complex is activated when ASFV DNA is bound, and it destroys a fluorescent ssDNA reporter contained in the test. The fluorophore on the ssDNA probe was released into the assay and detected by the fluorescence-sensing unit. Furthermore, using a blue LED or UV light illuminator, Ding et al. designed an all-in-one dual Cas12a (AIOD-CRISPR) test for SARS-CoV-2 detection that is simple, quick, ultrasensitive, selective, one-pot, and visible [43]. The Cas12a endonuclease was activated when the Cas12a-crRNA complexes bind the target sites, cleaving the FRET reporters and generating strong fluorescence signals. After incubation, the reaction mixture generated a super-bright fluorescence signal that could be observed clearly with a blue LED or UV light illuminator. The color of the reaction tube transition from orange-yellow to green could be seen with the naked eye even in the absence of stimulation under ambient light circumstances. All components for nucleic acid amplification and CRISPR-based detection are thoroughly mixed in a single, one-pot reaction system, obviating the requirement for separate pre-amplification and amplified product transfer.

Combined an automated and multiplexing CRISPR microfluidic chip with a custom-designed benchtop fluorometer for rapid- and low-volume virus detection, Qin et al., used Cas13a’s collateral RNA destruction after activation to establish an automated POC method for Ebola RNA detection [78]. Following automated microfluidic mixing and hybridization, nonspecific cleavage products of Cas13a are quickly analyzed by a patented integrated fluorometer, which is small in size and suitable for in-field diagnostics. The microfluidic chip is mounted on the fluorometer for in situ detection. By using a mobile phone camera to measure fluorescence within a compact device that includes low-cost laser illumination and collection optics. Fozouni et al. developed an amplification-free CRISPR/Cas13a assay for direct detection of SARS-CoV-2 from nasal swab RNA that can be read with a mobile phone microscope [76]. By combining multiple crRNAs to increase Cas13a activation then cleave any RNAs in the vicinity indiscriminately, detected using a fluorophore-quencher pair coupled by an RNA that fluoresces when active Cas13 cleaves it. The high sensitivity of mobile phone cameras, together with their connectivity, GPS, and data-processing capabilities, have made them attractive tools for point-of-care disease diagnosis in low-resource regions.

## 6. Conclusions and Outlook

A comprehensive understanding of various fluorescence readout types and variables affecting their analytical performance can facilitate superior experimental designs. This mini-review summarized different fluorescence readout types of the CRISPR/Cas system, as well as recent representative progress and applications. The fluorescence intensity was measured using dsDNA-specific fluorophores of cas9 and dcas9 based on binding activity. The fluorescence recovery was measured using FRET reporters for cas12, cas13, and cas14 based on trans cleavage activity.

With the advancement of CRISPR technology, the CRSIPR/Cas system, with its distinct characteristics, will provide infinite possibilities in the future. Despite significant advancements and appealing features currently, many challenges remain. It is currently difficult to achieve multiplexed detection in a single sample based on trans-cleavage activity by measuring the overall fluorescence intensity signal. According to a fluorescent digital principle similar to digital PCR, some researchers exploited the chip with adequate nanopores to achieve high-sensitivity digital CRISPR detection. Multiplexed detection is realized easily using space separation and fluorescent color coding. Furthermore, naked-eye detection can be achieved by test strips or simplified light sources, such as laser, LED, or smartphone light, which is appropriate for POCT settings with limited resources. However, CRISPR biosensors are dependent on signal amplification such as PCR, RPA, and LAMP presently. How to achieve amplification-free CRISPR detection using signal readout techniques is a major current challenge. The advancement of nanotechnology opens up new avenues to enrich the CRISPR signal readout library. More signal readout techniques combined with nanomaterials to achieve simpler and more sensitive analytical performance are a future trend for diverse applications of unique features of the CRISPR/Cas system. In addition, how to design the signal readout mode to realize multiplex detection is also a major challenge. Although challenges still remain, CRISPR/Cas-based nucleic acid detection will have broad applications, especially in the SARS-CoV-2 and other pathogenic diseases or cancers diagnosis.

## Figures and Tables

**Table 1:** Main features of CRISPR/Cas-based technology for diagnostic applications.

Effector	Detection Platform	Target	Amplification	Fluorescent Signal	Sensitivity	Applications	Signal Readout	References
**Cas9**		DNA/RNA	CAS-EXPAR	SYBR Green I	aM(10^−18^ M)	SNP genotype discrimination	Real-time fluorescence	[34]
	MOFs	DNA	SDA, RCA	FAM	40 CFU/mL (Colony-Forming Unit/mL)	Quantitative bacteria detection	Fluorescence spectroscopy	[35]
	CHP	DNA	SDA, RCA	FAM	aM	DNA and SNVs detection	Real-time fluorescence	[36]
	CRISDA	DNA	SDA	Cy5	2.5 aM	DNA and SNVs detection	Real-time fluorescence	[37]
	LEOPARD	DNA	qRT-PCR	TEX	aM	Virus detection	Real-time fluorescence	[38]
	FELUDA	DNA	RT-PCR	FAM	10 CPs/μL (copies/mL)	Virus and SNVs detection	Real-time fluorescence	[39]
	CADD	DNA	HCR; ELISA	FAM	aM	Viral, bacterial, and cellular DNA detection	Fluorescence spectroscopy	[40]
	PHC	DNA	HCR	FAM	0.025 pM	Multiplex virus detection	Fluorescence spectroscopy	[41]
**Cas12a**	HOLMES	DNA/RNA	PCR; RT-PCR	HEX	aM	Virus genotyping and human SNPs detection	Fluorescence spectroscopy	[31]
	DETECTR	DNA	RPA	FAM	aM	Virus detection	Real-time fluorescence	[42]
	AIOD-CRISPR	DNA	RT-RPA	FAM	5 CPs/μL	Virus detection	Real-time fluorescence	[43]
	deCOViD	DNA	RT-RPA	FAM	1 GE/μL (genome equivalent/μL)	Virus detection	Digital fluorescence	[44]
	CRISPR-FDS	DNA	RT-PCR; RT-RPA	FAM	2 CPs/sample	Virus detection	Digital fluorescence	[45]
	RADICA	DNA	RPA	FAM	10 pM (10^−12^ M)	Quantitative virus detection	Digital fluorescence	[46]
	WS-CRISPR	DNA	RT-DAMP	FAM	5 CPs/μL	Virus detection	Digital fluorescence	[47]
**Cas12b**	HOLMESv2	DNA/RNA	LAMP; RT-LAMP; Asymmetric PCR	HEX; FAM	aM	SNPs detection, RNA detection, DNA methylation detection	Real-time fluorescence	[48]
	STOP	DNA	RT-LAMP	HEX; FAM	33 CPs/mL	Virus detection	Real-time fluorescence	[29]
	CASdetec	DNA	RT-RAA	FAM	10^5^ CPs/mL	Virus detection	Real-time fluorescence	[49]
**Cas13a**	SHERLOCK	DNA/RNA	RPA	FAM	aM	Virus detecting, human DNA genotyping, cancer mutations	Real-time fluorescence	[21]
	Csm6	RNA	qRT-PCR	FAM	20 fM (10^−15^ M)	Virus detection	Real-time fluorescence	[50]
	SATORI	RNA	NONE	FAM	10 fM	Virus detection	Digital fluorescence	[51]
	CLISA	DNA	TMA	FAM	fM	VEGF detection	Real-time fluorescence	[52]
	CARMEN	DNA	PCR; RPA	FAM	10 CPs/μL	Multiplex virus and SNPs detection	Real-time fluorescence	[53]
	SHINE	DNA	RT-RPA	FAM	10 CPs/μL	Virus detection	Real-time fluorescence	[54]
**Cas13b**	SHERLOCKv2	DNA/RNA	RPA	FAM, TEX, Cy5, HEX	zM (10^−21^ M)	Multiplex detection	Real-time fluorescence	[55]
**Cas14a**	DETECTR	DNA	RPA	λex: 485 nm; λem: 535 nm	aM	SNP identification	Real-time fluorescence	[10]
**Cas14a1**	ATCas-RNA	RNA	TMA	FAM	aM	Nucleic acid detection	Real-time fluorescence	[28]

**Table 2:** Comparison of different fluorescent Cas 12 probes.

Method	Reporters	Reporters Sequences	LOD	References
DETECTR	ssDNA	5′6-FAM-TTATT-3′BHQ1	10^5^ copies/mL (with amplification)	[42]
HOLMES	ssDNA	5′6-HEX-NNNNNNNNNNNN-3′BHQ1	0.1 nM	[31]
NA	dsDNA	5′6-FAM-AGA ACC GAA TTT G TG TAG CTT ATC AGA CTG and CAG TCT GAT-AAG CTA CAC AAA TTC GGT TCT 3′IABkFQ	10 pM	[66]
NA	G4	5′6-FAM-T TAG GGT TAG GGT TAG GGT TAG GG-3′TAMRA	0.02 nM	[67]
G-CRISPR	G3	5′6-FAM-GGT TGG TGT GG-3′TAMRA	50 pM and 1000 copies/mL (with amplification)	[68]
NA	MB	5′6-Texas Red-TGG GAT AT CTT TAA TTT TAT TTT AAC AAG ATA TCC CA-3′BHQ	100 fM, 27 copies/mL (with amplification)	[65]

**Table 3:** Comparison of different fluorescent Cas 13 probes.

Method	Reporters	Reporters Sequences	LOD	Reference
SHINE	RNA	5′6-FAM-rUrUrUrUrUrUrUrUrUrUrUrUrUrU-3′Bio	10 CPs/μL	[54]
Cam6	RNA	5′6-FAM-TrCrUrArCrUrU-3′IABkFQ	fM	[50]
Sherlock	RNA	RNAse Alert v2, Thermo Scientific	aM	[21]
HUDSON	RNA	5′6-FAM-UUUUUUUUUUUUUU-3′Bio	0.9 aM (~1 cp/mL) in saliva 20 aM (10 cp/mL) in urine	[22]
vCAS	RNA	5′6-FAM-rUrUrUrUrU-3′BHQ1	fM	[69]
SATORI	RNA	5′6-FAM-rUrUrUrUrU-3′lABkFQ	fM	[51]

**Table 4:** Comparison of different fluorescent Cas 14 probes.

Method	Reporters	Reporters Sequences	LOD	Reference
ATCas-RNA	ssDNA	5′6-FAM-TTTTTTTTTTTT-3′BHQ1	aM	[28]
	RNA	5′6-FAM-UUUUU-3′BHQ1		
DETECTR	ssDNA	5′6-FAM-TTTTT-3′IABkFQ	aM	[70]
		5′6-FAM-TTTTTT-3′IABkFQ/		
		5′6-FAM-TTTTTTT-3′IABkFQ		
		5′6-FAM-TTTTTTTT-3′IABkFQ		
		5′6-FAM-TTTTTTTTT-3′IABkFQ		
		5′6-FAM-TTTTTTTTTT-3′IABkFQ		
		5′6-FAM-TTTTTTTTTTT-3′IABkFQ		
		5′6-FAM-TTTTTTTTTTTT-3′IABkFQ		

## Data Availability

Not applicable.
